# Valley-Related Multipiezo Effect in Altermagnet Monolayer V_2_STeO

**DOI:** 10.3390/ma18030527

**Published:** 2025-01-24

**Authors:** Yufang Chang, Yanzhao Wu, Li Deng, Xiang Yin, Xianmin Zhang

**Affiliations:** 1 Public Basic Department, Shenyang Conservatory of Music, Shenyang 110818, China; changyf537@foxmail.com; 2 Key Laboratory for Anisotropy and Texture of Materials (Ministry of Education), School of Material Science and Engineering, Northeastern University, Shenyang 110819, China; 2010235@stu.neu.edu.cn (Y.W.); 2210162@stu.neu.edu.cn (L.D.); 2210249@stu.neu.edu.cn (X.Y.)

**Keywords:** altermagnet, uniaxial strain, piezovalley, piezomagnetic, piezoelectric

## Abstract

The multipiezo effect realizes the coupling of strain with magnetism and electricity, which provides a new way of designing multifunctional devices. In this study, monolayer V_2_STeO is demonstrated to be an altermagnet semiconductor with a direct band gap of 0.41 eV. The spin splittings of monolayer V_2_STeO are as high as 1114 and 1257 meV at the valence and conduction bands, respectively. Moreover, a pair of energy degeneracy valleys appears at X and Y points in the first Brillouin zone. The valley polarization and reversion can be achieved by applying uniaxial strains along different directions, indicating a piezovalley effect. In addition, a net magnetization coupled with uniaxial strain and hole doping can be induced in monolayer V_2_STeO, presenting the piezomagnetic feature. Furthermore, due to the Janus structure, the inversion symmetry of monolayer V_2_STeO is naturally broken, resulting in the piezoelectric property. The integration of the altermagnet, piezovalley, piezomagnetic, and piezoelectric properties make monolayer V_2_STeO a promising candidate for multifunctional spintronic and valleytronic devices.

## 1. Introduction

Valley is a new degree of freedom for electrons locked within the momentum space [1,2] and is expected to be used in low-power electronic devices [3,4]. In recent years, a series of novel physical properties have been observed in two-dimensional valley materials, realizing the coordinated regulation of multiple effects. Valley-coupled light absorption is found in monolayer MoS_2_ with inversion symmetry breaking [5]. Intrinsic valley polarization and the corresponding reversion are achieved in ferromagnetic (FM) materials because of their natural breaking of center-inversion and time-reversal symmetries [6,7]. The valley-related Hall effect in antiferromagnetic (AFM) AuCl_3_ is regulated by changing its magnetic ground state through biaxial strain [8]. The altermagnet has been recently theoretically predicted and experimentally reported [9,10]; it combines the features of vanishing net magnetization in an antiferromagnet and natural spin splitting in a ferromagnet [11,12]. Up to date, reports about the valley physics in altermagnets are rare. Therefore, the valley-related properties in two-dimensional altermagnets are examined in detail in this study, aiming to expand the research scope of spintronics and valley electrons.

Strain is an effective method for regulating the physical properties of materials. A topological phase transition from the ferrovalley phase to the quantum anomalous Hall state is acquired in ferrovalley monolayers under biaxial strain [13,14]. The easy axis of monolayer GdBr_2_ transfers from in-plane to out-of-plane with a certain amount of compressive strain. The magnetic coupling state between FM and AFM can be manipulated in CrI_3_ by applying different strains [15]. The band gap tunability of transition metal dichalcogenides has been confirmed by applying a uniaxial tensile strain to three-terminal field effect transistors on a flexible substrate [16]. Consequently, the coupling of piezoelectricity and valley in the Janus monolayer VClBr is predicted to design multifunctional valleytronic and optoelectronic devices [17]. The feasibility of monolayer boron iodine (BI) with ferroelectricity, ultrahigh carrier mobility, and negative Poisson’s ratio has been demonstrated as a versatile platform for diverse nanoscale device applications [18]. Giant piezomagnetism and noncollinear spin current are reported in antiferromagnetic materials [19,20]. It will be interesting and meaningful to create the coupling between valley and strain, which may provide a new avenue to regulate valley polarization.

In this report, by first-principles calculations, the valley-related properties are systematically studied in monolayer V_2_STeO with and without strain. Monolayer V_2_STeO is proven to be an altermagnet semiconductor with a direct band gap of 0.41 eV and exhibits an in-plane magnetic anisotropy with a magnetic anisotropy energy (MAE) of 81 μeV/f.u. The spin splittings of monolayer V_2_STeO reach up to 1114 and 1257 meV at the valence and conduction bands, respectively. Moreover, a pair of energy degeneracy valleys appears at the X and Y points. The valley polarization and corresponding reversion can be realized in monolayer V_2_STeO by applying uniaxial strains along different directions, presenting a piezovalley feature. In addition, with the simultaneous action of uniaxial strain and hole doping, a net magnetization will be induced, indicating the piezomagnetic effect. Furthermore, due to the Janus structure, the inversion symmetry of monolayer V_2_STeO is natural broken, resulting in the piezoelectric property with a large strain tensor ($d_{31}$) of 0.36 pm/V. These studies indicate that monolayer V_2_STeO has potential applications in spintronics and valleytronics at room temperature. The spin–orbit coupling (SOC) effect is considered in the present work, and the piezoelectricity, Berry curvature, and spin splitting of monolayer V_2_STeO are also studied compared to the literature [21]. This paper is organized as follows: In Section 2, the computational method and details are provided. In Section 3, the stability property, magnetic feature, piezovalley effect, piezomagnetic effect, and piezoelectric effect of monolayer V_2_STeO are investigated. In Section 4, the conclusion is summarized.

## 2. Computational Method and Details

In this paper, first-principles calculations are performed using the Vienna Ab-initio Simulation Package (VASP) [22,23] based on the density functional theory (DFT). The projector-augmented wave (PAW) method [24,25] is used to describe the interaction of electrons and ions. The exchange–correlation function is treated by generalized gradient approximation (GGA) with the Perdew–Burke–Ernzerhof (PBE) functional [26,27]. Based on the convergence test (shown in Figure S1), the Monkhorst-Pack (MP) grid is chosen to be Γ-centered at 17 × 17 × 1 of the Brillouin zone in a sample [28]. The cut-off energy is set as 500 eV. The convergence criteria for atomic force and total energy correspond to 0.005 eV/Å and 10^−8^ eV, respectively. An 18 Å vacuum layer along the z direction is selected. The PBE + U_eff_ technique (U_eff_ = 4.3 eV) is used for the V-3d orbitals [29]. The phonon spectrum is obtained by the PHONOPY code [30] on the basis of the density functional perturbation theory [31]. A 4 × 4 × 1 supercell is adopted to calculate the ab initio molecular dynamic (AIMD) simulations at 300 K with a total of 6 ps (6000 fs) under a 3 fs time step [32]. The VASP data are processed by the VASPKIT code [33]. The localization function is created using the Wannier90 package [34]. The Berry curvature and anomalous Hall conductivity are calculated by the WannierTools package [35]. The elastic stiffness tensors $C_{ij}$ are calculated by using the strain–stress relationship (SSR) method. The piezoelectric stress tensors e_ij are calculated by the density functional perturbation theory (DFPT) method [36]. The $C_{ij}^{2D}/e_{ij}^{2D}$ are renormalized as $C_{ij}^{2D}=L_{z}C_{ij}^{3D}/{C_{ij}^{2D}=L_{z}e}_{ij}^{3D}/e_{ij}^{2D}$, where the $L_{z}$ is the length of a unit cell along the z direction.

## 3. Results and Discussion

### 3.1. Stability Property, Magnetic Feature, and Energy Band of Monolayer V_2_STeO

**Figure 1:**
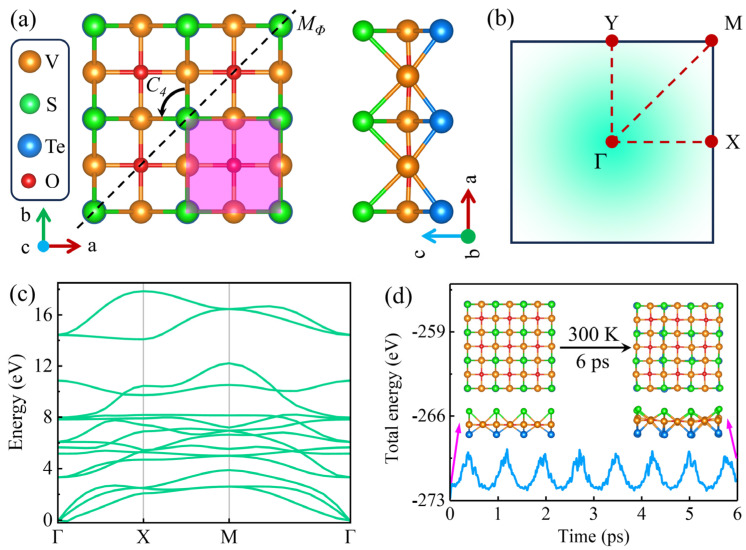
(**a**) Crystal structure of monolayer V_2_STeO. The pink square represents the unit cell. (**b**) The first Brillouin zone with high symmetry points. (**c**) Phonon spectrum of monolayer V_2_STeO. (**d**) Total energy fluctuation of the AIMD simulations for monolayer V_2_STeO. Insets are the structures of monolayer V_2_STeO before and after 6 ps.

Figure 1a shows the crystal structure of monolayer V_2_STeO, in which the V atom in the middle layer is sandwiched between the Se atom in the upper layer and the Te atom in the lower layer, presenting a Janus structure [20]. The pink region represents a unit cell, which exhibits a tetragonal lattice and contains two V, one S, one Te, and one O atoms. Monolayer V_2_STeO belongs to the P4mm space group and displays mirror (*M_Φ_*) and *C_4_* rotational symmetries. Figure 1b is the first Brillouin zone with high symmetry points, which corresponds to the tetragonal lattice. The optimized lattice constant of monolayer V_2_STeO is 4.08 Å. To indicate the stability, phonon spectra and AIMD simulations were further conducted. As shown in Figure 1c and Figure S2, all the phonon modes were positive throughout the Brillouin zone, indicating the dynamical stability of monolayer V_2_STeO [37,38]. As plotted in Figure 1d, the change in the total energy was very small during the AIMD simulations, and the structure maintained integrity after 6 ps, confirming the thermal stability of monolayer V_2_STeO [21,37,38].

**Figure 2:**
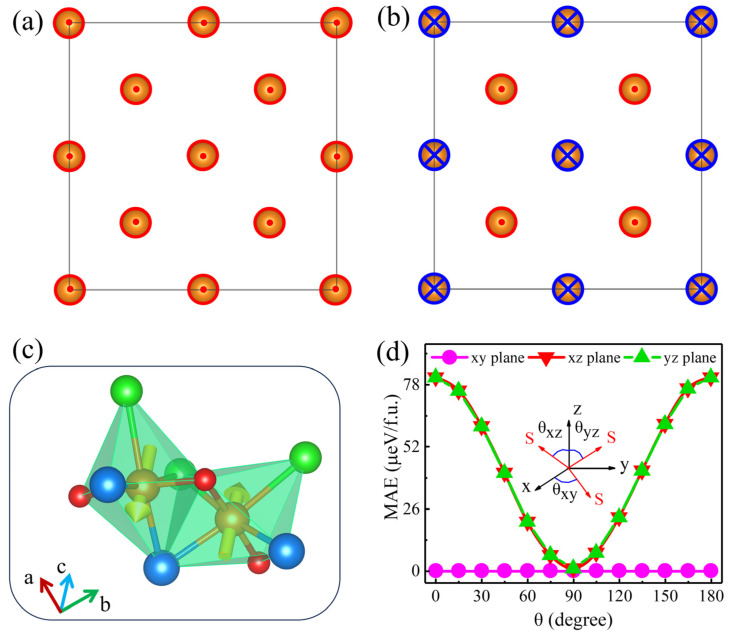
(**a**) FM and (**b**) AFM configurations of monolayer V_2_STeO. The red circle with a dot and blue circle with a fork denotes the spin-up and spin-down, respectively. (**c**) Magnetic structure of monolayer V_2_STeO, in which the yellow arrows indicate the spin alignment on the sublattice of the two V atoms. (**d**) The variation of MAE (μeV/f.u.) of monolayer V_2_STeO in the xy, xz, and yz planes as a function of the magnetization direction angle. The illustration shows the angle θ of rotation of the spin vector S between 0° and 180°.

The magnetic properties of monolayer V_2_STeO were also studied. To explore the magnetic ground state, the FM and AFM configurations were considered, as described in Figure 2a,b. The calculated energy difference between the two configurations (ΔE = E_FM_ − E_AFM_) was 383.21 meV/f.u. The acquired magnetic moment for each V atom was 2 μ_B_. Figure 2c shows the magnetic structure of monolayer V_2_STeO, in which the two V atoms have opposite spin directions. Moreover, the two V atoms can be related through combined translation and rotation operations rather than through the sample translation or reverse operations, indicating the altermagnetism of monolayer V_2_STeO [9,10]. Magnetic anisotropy is crucial for magnetic materials and can be evaluated by the magnetic anisotropy energy (MAE). The MAE is defined as follows: MAE = E[100] − Eθ, in which E[100] and E_θ_ are the energies of monolayer V_2_STeO with magnetization of the V atom along the [100] and θ directions, respectively, as plotted in the inset of Figure 2d. As shown in Figure 2d, the MAE remained 0 μeV/f.u. for different θ in the xy plane. Comparatively, as θ rotated in the xz and yz planes, the MAE varied from 0 μeV/f.u. to 81 μeV/f.u. The above results mean that monolayer V_2_STeO exhibits a lower energy with the magnetization of the V atom along the xy plane. Therefore, monolayer V_2_STeO presents an in-plane magnetic anisotropy with an MAE of 81 μeV/f.u.

**Figure 3:**
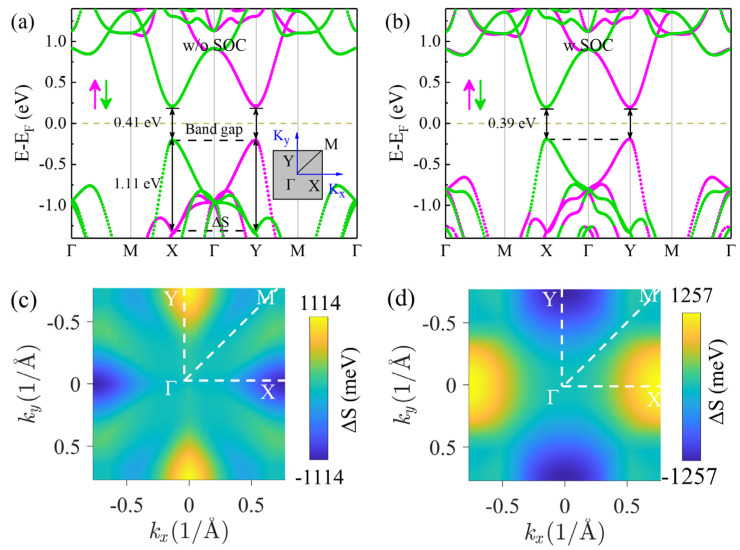
(**a**) Spin-resolved energy band of monolayer V_2_STeO without considering the spin–orbit coupling (SOC) effect. (**b**) Spin-resolved energy band of monolayer V_2_STeO considering the SOC effect. The magenta and green arrow denotes the spin-up and spin-down, respectively. (**c**) Spin splitting (ΔS = E_up (k) − E_down (k)) of the valence band for monolayer V_2_STeO throughout the first Brillouin zone. (**d**) The ΔS of the conduction band for monolayer V_2_STeO throughout the first Brillouin zone.

Furthermore, the electronic structure of monolayer V_2_STeO was investigated. As described in Figure 3a, without considering the spin–orbit coupling (SOC) effect, monolayer V_2_STeO behaved as a semiconductor with a direct band gap of 0.41 eV. Unexpectedly, the spin-up and spin-down states were not overlapping, and the spin splitting (ΔS = E_up_(k) − E_down_(k)) energy at X points on the valence band reached up to 1.11 eV. This is far larger than that of 100 meV resulting from SOC in compounds with heavy atoms [39]. It differs from the traditional AFM materials [8], in which the spin-up and spin-down states are completely overlapped. Moreover, a pair of energy valleys occurs at the X and Y high symmetry points with the same energies. As displayed in Figure 3b, considering the SOC effect, monolayer V_2_STeO retained a semiconductor characteristic and a spin-splitting feature. In addition, the two valleys remained in the energy degeneracy state. This is different from the case of ferrovalley materials, in which valley polarization will be induced with the SOC effect and intrinsic magnetic exchange interaction [6,7]. The spin splitting throughout the first Brillouin zone was further studied in detail. Figure 3c shows the spin splitting of the valence band for monolayer V_2_STeO throughout the first Brillouin zone. It can be found that the minimum and maximum spin splitting appeared at X and Y with a value of −1114 meV and 1114 meV, respectively. Furthermore, the spin splitting was diagonally antisymmetric in the entire Brillouin zone, resulting in a zero sum for all these values. As shown in Figure 3d, the spin splitting of the conduction band was also diagonally symmetric, and the maximum was 1257 meV. This spin splitting has been observed in the altermagnetic materials MnTe, CrSb, and Rb_1-*δ*_V_2_Te_2_O in an experiment by ARPES [12,40,41].

### 3.2. Piezovalley Effect of Monolayer V_2_STeO

**Figure 4:**
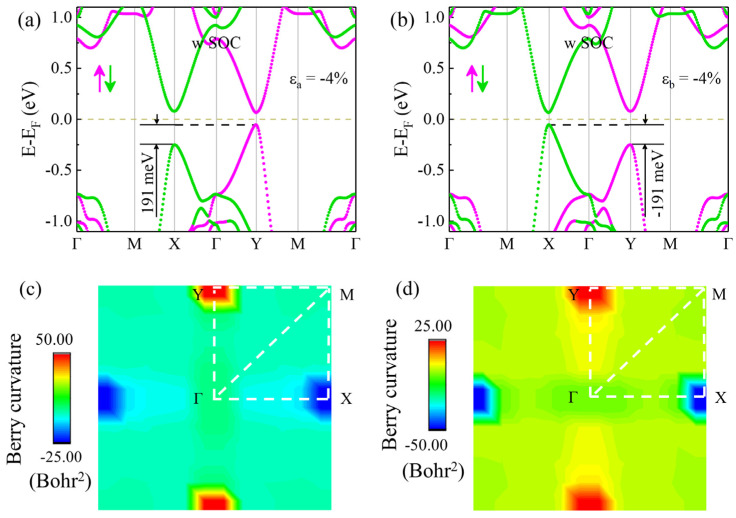
(**a**) Spin-resolved energy band with SOC effect for V_2_STeO monolayer under −4% uniaxial strains along the a direction. (**b**) Spin-resolved energy band with SOC effect for V_2_STeO monolayer under −4% uniaxial strains along the b direction. The magenta and green arrow denotes the spin-up and spin-down, respectively. (**c**) Berry curvatures of V_2_STeO monolayer under −4% uniaxial strains along the a direction. (**d**) Berry curvatures of V_2_STeO monolayer under −4% uniaxial strains along the b direction.

If a uniaxial strain is applied along the a or b direction, the *M_Φ_* symmetry of monolayer V_2_STeO will be broken. At the same time, the rotational symmetry of monolayer V_2_STeO will become *C_2_* from *C_4_*. This will also lift the degeneracy between the X and Y valleys, which can realize a valley polarization in present monolayer V_2_STeO. In order to verify the above suspicion, the monolayer V_2_STeO at −4% strains along the a and b directions was used as an example for the relevant investigation, as shown in Figure 4. The magnitude of uniaxial strain is defined as ε = (a − a_0_)/a_0_ × 100%, where a and a_0_ are the lattice constants of monolayer V_2_STeO with and without strains, respectively. When a −4% strain along the a direction was applied, the valley polarization of monolayer V_2_STeO was 191 meV, which is larger than valley polarization of 120 meV in monolayer MoS2 [42]. It is large enough to overcome the 24 meV energy of room-temperature thermal noise [37], which is a random electrical signal generated by the thermal motion of carriers in materials [4]. On applying a −4% strain along the b direction, as shown in Figure 4b, monolayer V_2_STeO also presented a −191 meV valley polarization. Therefore, valley polarization and reversion can be realized by applying different uniaxial strains in monolayer V_2_STeO.

To further demonstrate the polarization and reversion of valley, the Berry curvature of monolayer V_2_STeO was also studied, which can be calculated based on the Kubo formula [43,44]:

(1) $$\Omega_{z}\left(k\right)=-\sum_{n}\sum_{n\neq m}f_{n}\left(k\right)\frac{2Im\langle \psi_{nk}\left|{\hat{\upsilon }}_{x}\right|\psi_{mk}\rangle \langle \psi_{mk}\left|{\hat{\upsilon }}_{y}\right|\psi_{nk}\rangle }{{\left(E_{nk}-E_{mk}\right)}^{2}}$$

where ƒ_n_(**k**) is Fermi–Dirac distribution function with **k** being the electron wave vector; E_n**k**_ and E_m**k**_ are the eigenvalues of the Bloch wave functions ψ_n**k**_ and ψ_m**k**_; ${\hat{\upsilon }}_{x}$ and ${\hat{\upsilon }}_{y}$ are velocity operators of the Dirac electrons; n and n′ are the band indexes. Figure 4c displays the Berry curvature of monolayer V_2_STeO with a −4% strain along the a direction. The Berry curvatures of the X and Y valleys were −25.00 and 50.00 Bohr^2^, respectively. The two values had opposite signs and unequal absolute values, further indicating the valley contrasting characteristic of monolayer V_2_STeO. As drawn in Figure 4d, for a −4% strain along the b direction, the Berry curvature of the X and Y valleys became −50.00 and 25.00 Bohr^2^, respectively. The sign of the Berry curvature for the X and Y valleys did not change, while the absolute value of the X valley became smaller than that of the Y valley, which indicates the valley polarization is reversed. Therefore, the valley polarization of monolayer V_2_STeO can be regulated by applying uniaxial strains along different directions. Due to the coupling of valley polarization and uniaxial strain, monolayer V_2_STeO presents a piezovalley effect.

**Figure 5:**
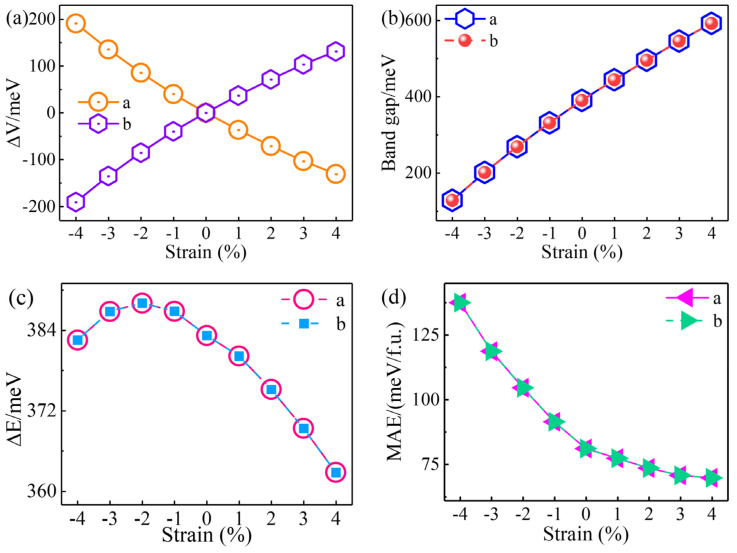
(**a**) Valley polarization of monolayer V_2_STeO under different uniaxial strains along the a and b directions. (**b**) Band gap of monolayer V_2_STeO under different uniaxial strains along the a and b directions. (**c**) Energy difference (ΔE) between FM and AFM states of monolayer V_2_STeO under different uniaxial strains along the a and b directions. (**d**) The MAE of monolayer V_2_STeO under different uniaxial strains along the a and b directions.

Under the action of uniaxial strain, due to the changes in the crystal structure, the valley polarization, band gap, energy difference (ΔE), and MAE will be inevitably affected. Therefore, we further studied these properties in monolayer V_2_STeO under different strains in detail. As shown in Figure 5a, for the strain along the a direction, the valley polarization decreased as the strain changed from −4% to 4% and became a negative value from the positive value for tensile strains. The corresponding energy bands are provided in Figure S3. On applying a strain along the b direction, monolayer V_2_STeO also exhibited the valley polarization property. The difference is that, under the same strain value, the valley polarization sign in the b strain was opposite to that of the a strain, indicating that the valley polarization was mutually reversed in the two strains. The above results indicate that the valley polarization of monolayer V_2_STeO can be efficiently adjusted by applying a uniaxial strain along the a and b directions, respectively. As plotted in Figure 5b and Figure S3, during the −4% ~ 4% uniaxial strains, monolayer V_2_STeO retained the semiconductor characteristic, and the band gap increased gradually. Moreover, among the uniaxial strains, the anomalous valley Hall conductivity of monolayer V_2_STeO was non-integer and did not possess quantum characteristics, as shown in Figure S4. Therefore, monolayer V_2_STeO was always topologically trivial. As displayed in Figure 5c, under the −4% ~ 4% strains, the energy difference (ΔE) of monolayer V_2_STeO first decreased and then increased and always maintained a positive value, indicating the AFM coupling under all of the strains. Moreover, the MAE of monolayer V_2_STeO decreased gradually from −4% to 4% uniaxial strains and retained a positive value, proving the in-plane magnetic anisotropy.

### 3.3. Piezomagnetic Effect of Monolayer V_2_STeO

**Figure 6:**
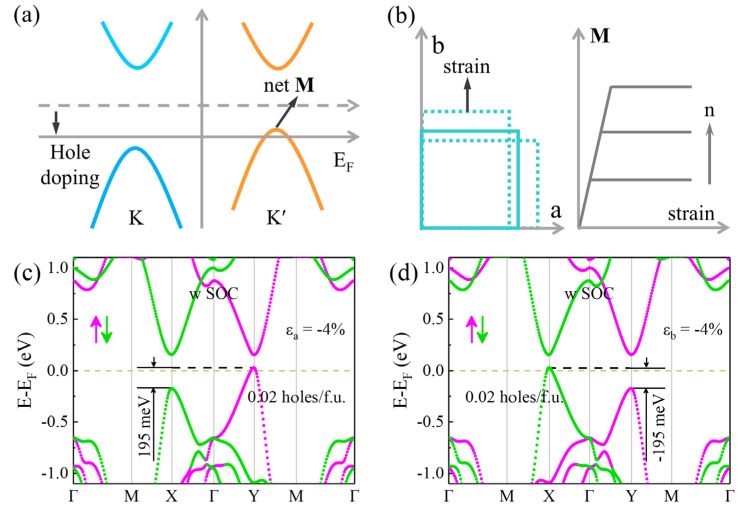
(**a**) Schematic of the induced net magnetization (**M**) by hole doping in monolayer V_2_STeO with a valley polarization under a certain strain. (**b**) Schematic of the strain and the dependence of the induced **M** on the strain and hole-doping amount in monolayer V_2_STeO. (**c**) Spin-resolved energy band of monolayer V_2_STeO with doping 0.02 holes/f.u. under −4% uniaxial strains along the a direction. (**d**) Spin-resolved energy band of monolayer V_2_STeO with doping 0.02 holes/f.u. under −4% uniaxial strains along the b direction. The magenta and green arrow denotes the spin-up and spin-down, respectively.

For a magnetic material, its magnetic moment is equal to the difference of the integral between the spin-up and spin-down states in the region below the Fermi level. Therefore, a net magnetization (**M**) can be induced in monolayer V_2_STeO with a certain uniaxial strain by adjusting the Fermi level crossing one valley through hole doping, as shown in Figure 6a. Under this case, for a certain strain, the induced **M** increased with the improvement in the hole-doping concentration and ultimately achieved saturation, as shown in Figure 6b. Similarly, for a given hole-doping amount, the induced **M** first enlarged monotonically with the rise in the strain and then gradually reached its maximum value. To verify the above speculation, the energy band of monolayer V_2_STeO with uniaxial strain and hole doping was first studied. As shown in Figure 6c, on applying a −4% strain and doping 0.02 holes/f.u., monolayer V_2_STeO retained the valley polarization feature with a value of 195 meV. Moreover, the Y valley from the spin-up state crossed the Fermi level, while the X from the spin-down state was still located below the Fermi level. It results in a 100% spin-up polarization in monolayer V_2_STeO, which is crucial for the application of spintronics devices. Comparatively, on applying a −4% strain and doping 0.02 holes/f.u., the valley polarization of monolayer V_2_STeO reversed to −195 meV, and the X valley from spin-down state crossed the Fermi level, resulting in a 100% spin-down polarization, as exhibited in Figure 6d. The above electric structure is consistent with that described in Figure 6a.

**Figure 7:**
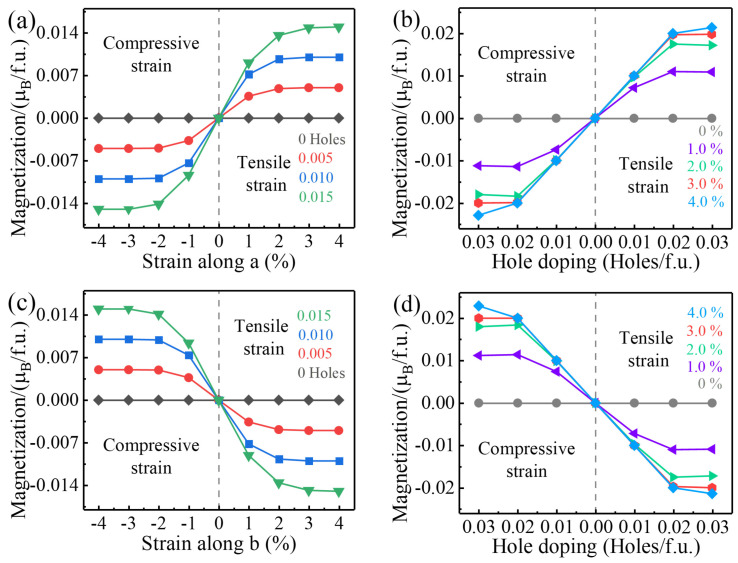
(**a**) Corresponding **M** for different strains along the a direction under a certain hole-doping amount. (**b**) Corresponding **M** for different hole-doping amounts with a certain strain along the a direction. (**c**) Induced **M** under various strains along the b direction under a given hole-doping amount. (**d**) Induced **M** under various hole-doping amounts with a given strain along the b direction.

Furthermore, the induced **M** of monolayer V_2_STeO on applying different uniaxial strains and doping various holes/f.u. was further studied in detail. As shown in Figure 7a, for a given amount of hole doping, the corresponding **M** in monolayer V_2_STeO enlarged linearly with increases in the strain at small strains and gradually reached a maximum value for larger strains. As plotted in Figure 7b, with certain strains, the net **M** also first increased under a low hole-doping concentration and finally realized saturation. On applying a uniaxial strain along the b direction, as exhibited in Figure 7c, the occurring net **M** was also positively correlated with the strength of the applied strain and gradually became constant at the end of the process. Notably, the net **M** had an equal value but presented an opposite sign for the same strain along the a and b directions, respectively. Similarly, for a defined strain, the triggering net **M** in monolayer V_2_STeO was proportional to the number of holes for a limited doping concentration and finally acquired the largest value, as shown in Figure 7d. The above phenomenon is the same as that in our analysis in Figure 6b. Therefore, net **M** can be induced in monolayer V_2_STeO by applying uniaxial strains and doping holes. Due to the coupling of producing net **M** and uniaxial strain, monolayer V_2_STeO presents a piezomagnetic property. Notably, 0.03 Holes/f.u. doping in the present monolayer V_2_STeO corresponds to a carrier doping density of around 1.74 × 10^12^ Holes/cm^2^, which can be easily achieved through available gate techniques [45,46].

### 3.4. Piezoelectric Effect of Monolayer V_2_STeO

**Figure 8:**
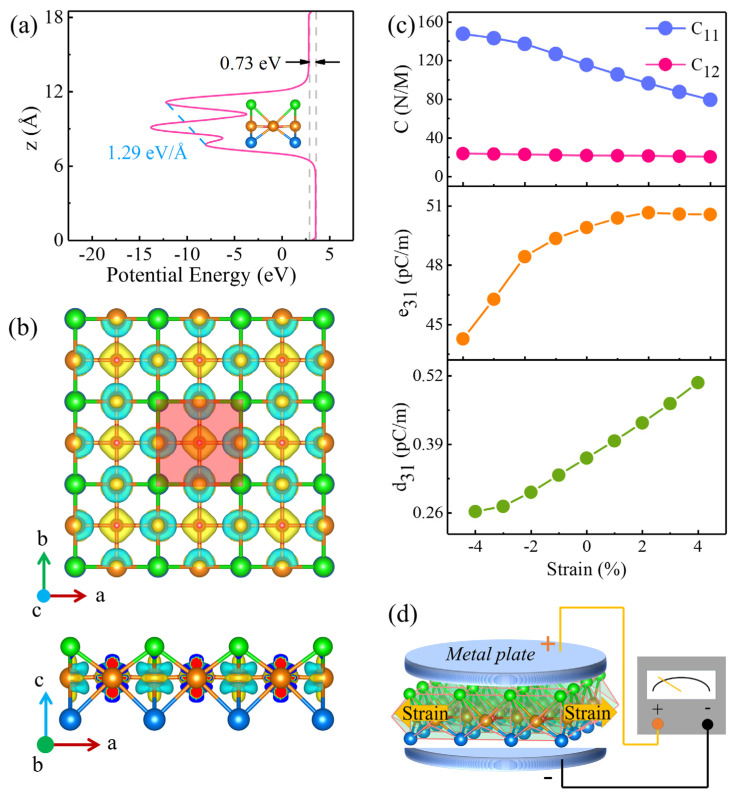
(**a**) Plane-averaged electrostatic potential of monolayer V_2_STeO along the z direction. (**b**) Differential charge density diagram of monolayer V_2_STeO. The red square region denotes the unit cell. The yellow area is the region that gained electrons, and the cyan area is the region that lost electrons. (**c**) The C ($C_{11}$ and $C_{12}$), $e_{31}$, and $d_{31}$ of monolayer V_2_STeO under different biaxial strains. (**d**) Schematic diagram of the mechanism for the piezoelectric effect in monolayer V_2_STeO.

The piezoelectric effect is exhibited in materials without inversion symmetry, which will induce an electrical polarization and realize the coupling between the strain as well as the polarization strength [47,48,49]. Due to the Janus structure of monolayer V_2_STeO, the horizontal mirror symmetry of monolayer V_2_STeO is naturally broken, resulting in an out-of-plane piezoelectricity. To verify the above guess, the plane-averaged electrostatic potential, differential charge density, and elastic-related constants were systematically studied. As shown in Figure 8a, an electrostatic potential difference with the value of 0.73 meV was generated between the vacuum levels of the top and bottom layers in monolayer V_2_STeO, indicating the existence of an out-of-plane dipole moment. Moreover, a 1.29 eV/Å intrinsic electric field was present in monolayer V_2_STeO. This is caused by the nonuniform distribution of charge density between the S atoms in the top layer and the Te atoms in the bottom layer, which can be explained by the differential charge density. As exhibited in Figure 8b, the V atoms lost electrons, while the S, Te, and O atoms gained electrons. Moreover, the S atom gained more electrons than the Te atom, resulting in a vertical electrical polarization. The intrinsic electric field and the out-of-plane dipole moment are the typical characteristics of a piezoelectric material [47,48].

The piezoelectricity can be described by the third-rank piezoelectric stress tensor ($e_{ijk}$) and the strain tensor ($d_{ijk}$), which can be expressed as follows [49,50]:

(2) $$e_{ijk}=\frac{{\partial P}_{i}}{{\partial \varepsilon }_{jk}}=e_{ijk}^{elc}+e_{ijk}^{ion}$$

(3) $$d_{ijk}=\frac{{\partial P}_{i}}{{\partial \sigma }_{jk}}=d_{ijk}^{elc}+d_{ijk}^{ion}$$

where i, j, and k represent the x, y, and z Cartesian directions; $P_{i}$ is polarization vector; $\varepsilon_{jk}$ is strain; $\sigma_{jk}$ is stress; and $e_{ijk}^{elc}$ and $e_{ijk}^{ion}$ denote the electronic and ionic contribution components, respectively. The $e_{ijk}$ is related with $d_{ijk}$ by the elastic tensor $C_{mnjk}$:

(4) $$e_{ijk}=\frac{{\partial P}_{i}}{{\partial \varepsilon }_{jk}}=\frac{{\partial P}_{i}}{{\partial \sigma }_{mn}}\times \frac{{\partial \sigma }_{mn}}{{\partial \varepsilon }_{jk}}=d_{imn}C_{mnjk}$$

Based on the Voigt notation, the number of independent tensors of Equation (4) is reduced as follows:

(5) $$\left(\begin{array}{ccc} 0 & 0 & 0\\ 0 & 0 & 0\\ e_{31} & e_{31} & 0 \end{array}\right)=\left(\begin{array}{ccc} 0 & 0 & 0\\ 0 & 0 & 0\\ d_{31} & d_{31} & 0 \end{array}\right)\left(\begin{array}{ccc} C_{11} & C_{12} & 0\\ C_{12} & C_{11} & 0\\ 0 & 0 & C_{66} \end{array}\right)$$

The out-of-plane piezoelectric polarization ($e_{31}/d_{31}\neq 0$) can be induced, with the application of an in-plane uniaxial strain. By Equation (5), $d_{31}$ can be calculated to be equal to the following:

(6) $$d_{31}=\frac{e_{31}}{C_{11}+C_{12}}$$

The $e_{31}$ was calculated to be 49.90 pC/m based on the DFPT method [36]. Then, the $d_{31}$ was estimated to be 0.36 pm/V, which is larger than the corresponding values of monolayer CrSSiN_2_ (0.28 pm/V) [51] and MoSiN_3_H (0.058 pm/V) [52], while it is smaller than the corresponding values of 3R-MoS_2_ flakes (1.64 pm/V) [53]. The effect of biaxial strain on the piezoelectric properties of V_2_STeO monolayer was also studied. As shown in Figure 8c, the C ($C_{11}$ and $C_{12}$) decreased from −4% to 4% biaxial strains, while the $e_{31}$ and $d_{31}$ increased under −4% ~ 4% biaxial strains. Therefore, the polarization strength of monolayer V_2_STeO can be effectively regulated by the strain. As a result, monolayer V_2_STeO presents a good piezoelectric effect. Figure 8d provides a schematic of the piezoelectric voltage generated by the monolayer V_2_STeO. Additionally, it is noted that monolayer VSe_2_ was proposed by the first-principles calculations [6] before it was grown by the chemical vapor deposition method [54]. Bulk CsV_2_Se_2-x_O and V_2_Se_2_O have been fabricated using solid-state reactions [55]. Therefore, monolayer V_2_STeO could be grown by the chemical vapor deposition method or by mechanical exfoliation from the bulk material. Therefore, we expect that the present monolayer V_2_STeO will be grown by experimental scientists.

## 4. Conclusions

In conclusion, the magnetic property, electric structure, and piezo effect of monolayer V_2_STeO were systematically studied. Monolayer V_2_STeO was demonstrated to be an altermagnet with an in-plane magnetic anisotropy and behaved as a semiconductor with a direct band gap of 0.41 eV. The spin splittings of monolayer V_2_STeO reached up to 1114 and 1257 meV at the valence and conduction bands, respectively. Moreover, a pair of energy degeneracy valleys appeared at X and Y points. The valley polarization and its reversion would be achieved in monolayer V_2_STeO by applying uniaxial strains along different directions, presenting a piezovalley feature. In addition, with the simultaneous action of uniaxial strain and hole doping, a net **M** will be induced, indicating the piezomagnetic effect. Furthermore, due to the Janus structure, the inversion symmetry of monolayer V_2_STeO was naturally broken, resulting in the piezoelectric property with a large strain tensor ($d_{31}$) of 0.36 pm/V. Therefore, the altermagnet monolayer V_2_STeO exhibited a multipiezo effect, which provides a good opportunity for designing multifunctional devices.

## Data Availability

The original contributions presented in this study are included in the article/Supplementary Material. Further inquiries can be directed to the corresponding author.

## Supplementary Materials

The following supporting information can be downloaded at: https://www.mdpi.com/article/10.3390/ma18030527/s1, Figure S1: Convergence tests of Monkhorst-Pack grid density about monolayer V2STeO; Figure S2: The original data of phonon spectra for monolayer V2STeO; Figure S3: Spin-resolved energy bands of monolayer V2STeO under different uniaxial strains along a direction; Figure S4: Anomalous valley Hall conductivity of monolayer V2STeO under different uniaxial strains along a direction.
