# Supplementary material for: Valley-Related Multipiezo Effect in Altermagnet Monolayer V2STeO
Source: Materials (Basel). 2025 Jan 24;18(3):527. doi: 10.3390/ma18030527 (PMC11818174; doi:10.3390/ma18030527)
Supplement: Supplementary file 1 [file materials-18-00527-s001.zip › materials-3419734-supplementary.pdf]

Supplemental material

Valley-related multipiezo effect in altermagnet monolayer V_2STeO

Yufang Chang[a], Yanzhao Wu[b], Li Deng[b], Xiang Yin[b], and Xianmin Zhang[b]*

[a]Public Basic Department, Shenyang Conservatory of Music, Shenyang 110818, China

[b]Key Laboratory for Anisotropy and Texture of Materials (Ministry of Education), School of Material Science and Engineering, Northeastern University, Shenyang, 110819, China

The Monkhorst-Pack grid density is optimized by conducting a convergence test. As shown in Figure S1, as the Monkhorst-Pack grid density reaching 17×17×1, the energy of monolayer V_2STeO reaches the lowest value and becomes stable. Therefore, the Monkhorst-Pack grid density is set as 17 × 17 × 1.

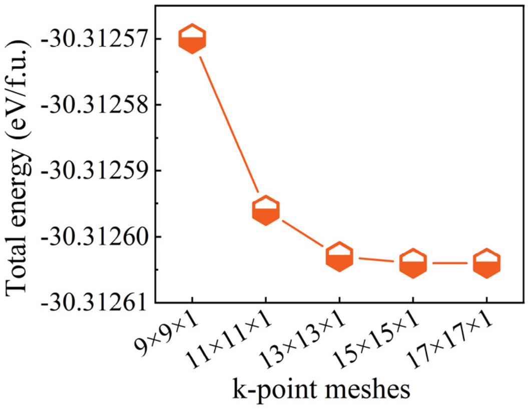

Figure S1. Convergence tests of Monkhorst-Pack grid density about monolayer V_2STeO.

0	-0.08858	-0.08858	-0.02624	3.30599	3.30599	5.14703	5.64271	6.06219	6.06219	7.82334	7.82334	7.96274	10.85639	14.42509	14.42509
0.01362	0.49673	0.83934	-0.07683	3.33476	3.37651	5.14216	5.62937	6.16242	6.06391	7.82621	7.85439	7.9808	10.8116	14.55711	14.41529
0.02723	1.01817	1.57719	0.29392	3.42364	3.58606	5.14991	5.60447	6.42845	6.06712	7.83424	7.92221	8.04699	10.68482	14.9297	14.38692
0.04084	1.47182	2.11823	0.5472	3.57959	3.89769	5.21242	5.58394	6.81908	6.06619	7.84588	7.99518	8.16393	10.49836	15.47602	14.343
0.05446	1.84064	2.45326	0.82656	3.80868	4.19652	5.33504	5.60985	7.27896	6.05212	7.85916	8.07897	8.32503	10.28675	16.10025	14.28835
0.06807	2.114	2.62858	1.12323	4.10463	4.39692	5.38165	5.81088	7.63706	6.0136	7.87227	8.12564	8.67299	10.08904	16.70328	14.22916
0.08169	2.3001	2.68185	1.4223	4.44275	4.52237	5.3501	6.1272	7.76103	5.93852	7.88385	8.11112	9.25506	9.9346	17.20755	14.1725
0.0953	2.41717	2.63986	1.70692	4.78974	4.60847	5.30396	6.45287	7.68338	5.81613	7.89294	8.12168	9.90184	9.77343	17.57016	14.12552
0.10892	2.48121	2.53684	1.95128	5.1161	4.66325	5.2663	6.74101	7.50502	5.63946	7.8988	8.15559	10.28875	9.74695	17.78136	14.09445
0.12253	2.50157	2.46122	2.07327	5.37728	4.68251	5.25174	6.88955	7.38782	5.43065	7.90083	8.17117	10.44078	9.72649	17.84968	14.08358
0.12253	2.50157	2.46122	2.07327	5.37728	4.68251	5.25174	6.88955	7.38782	5.43065	7.90083	8.17117	10.44078	9.72649	17.84968	14.08358
0.13615	2.60434	2.39953	2.0979	5.4638	4.67985	5.23173	6.90946	7.31572	5.46484	7.93458	8.17205	10.41947	9.74644	17.80752	14.1949
0.14976	2.7618	2.36116	2.16486	5.67828	4.6865	5.19151	6.96458	7.15305	5.54402	7.98143	8.17447	10.39187	9.80748	17.68612	14.49377
0.16338	2.94914	2.3591	2.25694	5.93428	4.71604	5.14692	7.03735	6.96393	5.64125	7.98186	8.17641	10.43912	9.9085	17.50013	14.90165
0.17699	3.15383	2.38745	2.35465	6.16375	4.76564	5.10136	7.09504	6.78884	5.73194	7.91938	8.17317	10.63137	10.0392	17.27191	15.33752
0.19061	3.36151	2.43671	2.44271	6.34048	4.82568	5.05713	7.10847	6.67209	5.77865	7.80136	8.16305	10.97535	10.18118	17.02876	15.73711
0.20422	3.5562	2.49461	2.51251	6.46812	4.88549	5.01867	7.076	6.65636	5.74558	7.64449	8.15134	11.4032	10.31454	16.79977	16.05811
0.21784	3.71943	2.54799	2.56123	6.55753	4.93556	4.99224	7.01918	6.73425	5.65121	7.46784	8.14344	11.81208	10.42245	16.61253	16.28125
0.23145	3.8302	2.58526	2.58943	6.61484	4.9686	4.98141	6.96613	6.85825	5.55665	7.29453	8.14	12.10439	10.4924	16.48992	16.40732
0.24507	3.86972	2.5986	2.5986	6.63599	4.98012	4.98012	6.94379	6.94379	5.51711	7.19374	8.13919	12.21041	10.51662	16.44726	16.44726
0.24507	3.86972	2.5986	2.5986	6.63599	4.98012	4.98012	6.94379	6.94379	5.51711	7.19374	8.13919	12.21041	10.51662	16.44726	16.44726
0.26432	3.79343	2.60335	2.54673	6.60349	5.00583	4.93382	6.98348	6.79626	5.58891	7.36898	8.14034	11.9994	10.4686	16.38763	16.5088
0.28358	3.60476	2.60957	2.38135	6.6033	5.07331	4.81064	7.05651	6.56669	5.68614	7.65124	8.13902	11.42144	10.3352	16.219	16.62797
0.30283	3.37431	2.59477	2.09432	6.73583	5.16672	4.61166	7.13015	6.28975	5.62681	7.93192	8.11749	10.60314	10.14636	15.96825	16.67283
0.32208	3.13301	2.52437	1.71292	6.80368	5.27313	4.32318	7.28032	5.93703	5.45808	8.17117	7.98858	9.63002	10.05334	15.66735	16.53357
0.34134	2.86752	2.34818	1.28952	6.69177	5.37734	3.96942	7.49674	5.46267	5.29432	8.1954	7.68015	8.90778	10.04599	15.34139	16.16699
0.36059	2.53404	1.99881	0.87098	6.49944	5.47813	3.64419	7.66719	4.79628	5.18248	8.08668	7.09431	8.51234	10.24722	15.0121	15.62121
0.37985	2.02062	1.44521	0.49051	6.29517	5.55681	3.43362	7.76691	4.03257	5.134	7.98725	6.55375	8.21162	10.53476	14.7148	15.03534
0.3991	1.15355	0.7378	0.14593	6.12958	5.61561	3.33376	7.81161	3.48255	5.13614	7.89331	6.19671	8.00631	10.76818	14.50255	14.58997
0.41836	-0.08858	-0.08858	-0.02624	6.06219	5.64271	3.30599	7.82334	3.30599	5.14703	7.82334	6.06219	7.96274	10.85639	14.42509	14.42509

Figure S2. The original data of phonon spectra for monolayer V_2STeO.

As shown in Figure S3(a), under -4% uniaxial compressive strain along a direction, the energy value of X point in the valence band is smaller than that of Y point, which means that monolayer V_2STeO presents a valley polarization of 191 meV. As applied -3.0% ~ 0.0% uniaxial compressive strains, monolayer V_2STeO also keeps the valley polarization state, and the valley polarization decreases with the decreases of compressive strain, as shown in Figures S3(b)-S3(e). Therefore, valley polarization can be realized in monolayer V_2STeO using uniaxial strain. As applied 1.0% ~ 4.0% uniaxial tensile strains along a direction, monolayer V_2STeO also exhibits a valley polarization at the X point, and the polarization value becomes larger with increases the tensile strain, as shown in Figures S3(f)-S3(i). Therefore, valley polarization and corresponding reversion can be achieved by applying a uniaxial compressive and tensile strains, respectively. Notably, during the strains, monolayer V_2STeO always behaves as a semiconductor, and the band gap increases from -4% to 4% strains.

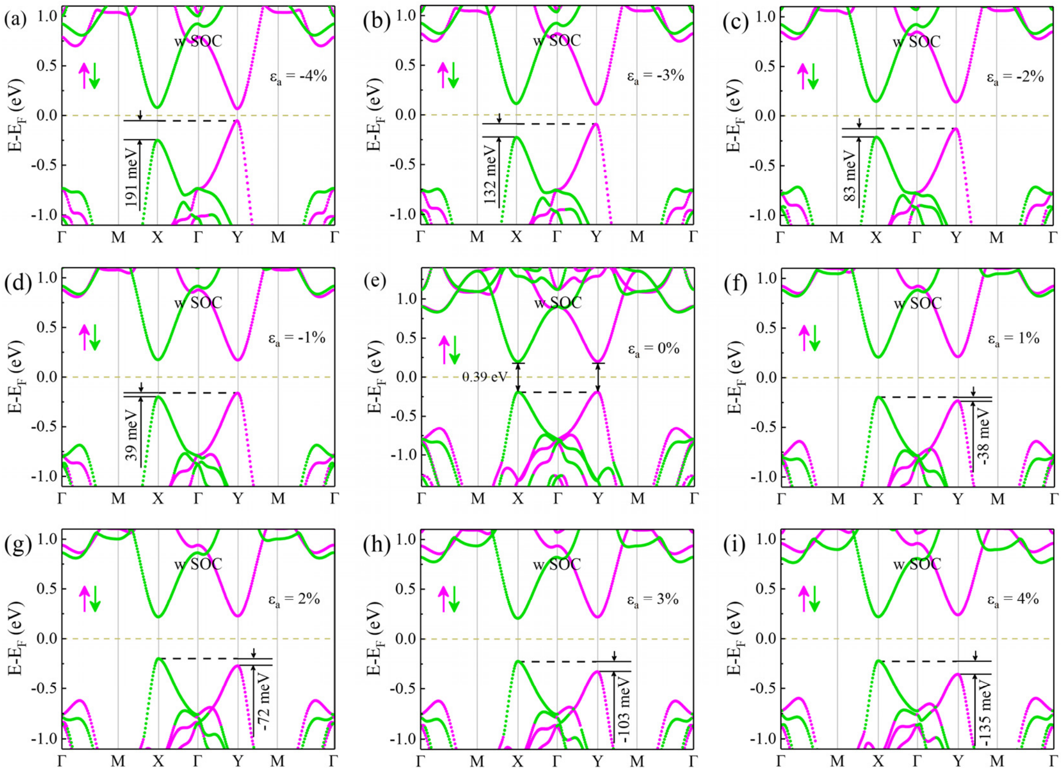

Figure S3. Spin-resolved energy bands of monolayer V_2STeO under different uniaxial strains along a direction.

Figure S4 shows the anomalous valley Hall conductivity of monolayer V_2STeO under different uniaxial strains along a direction. During the uniaxial strains, the anomalous valley Hall conductivity of monolayer V_2STeO is non-integer and does not possess quantum characteristics. Therefore, monolayer V_2STeO is always topological trivial.

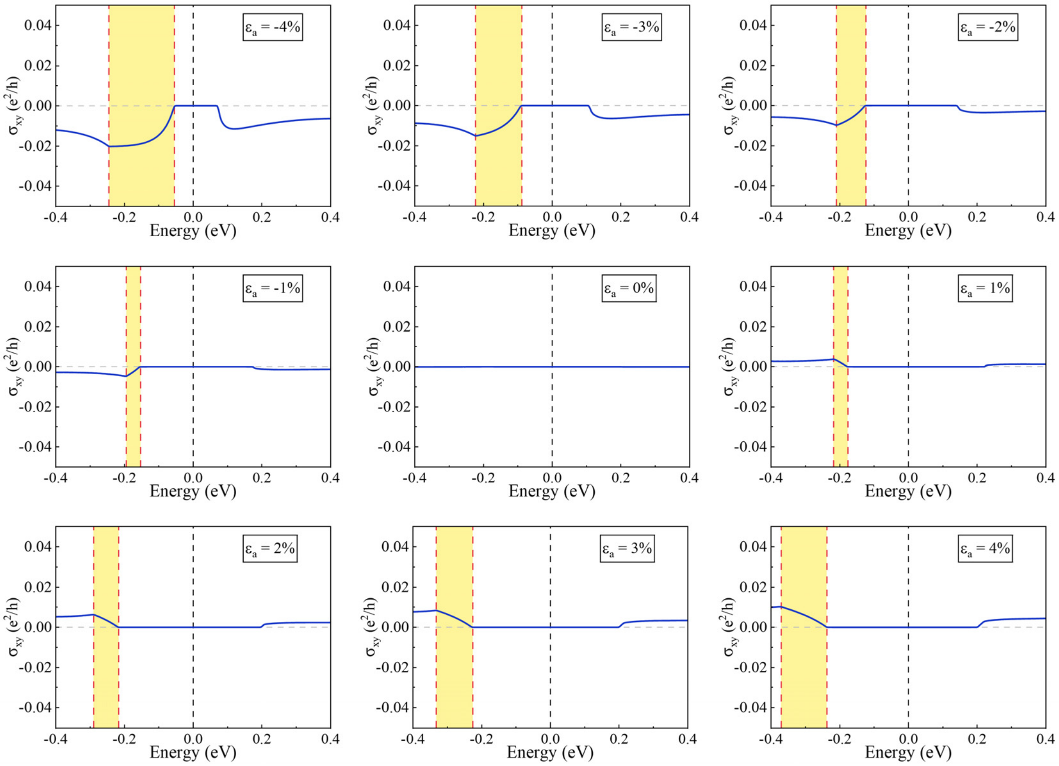

Figure S4. Anomalous valley Hall conductivity of monolayer V_2STeO under different uniaxial strains along a direction. The two vertical dashed lines (yellow region) denote the two valley extrema.
